# Beyond the spatial fix: towards a finance-sensitive reading of the Belt and Road in Serbia

**DOI:** 10.1080/23792949.2023.2200546

**Published:** 2023-05-24

**Authors:** Imogen Taotao Liu

**Affiliations:** Department of Political Science and Public Administration, Vrije Universiteit Amsterdam, Amsterdam, The Netherlands

**Keywords:** Belt and Road Initiative (BRI), financialization, infrastructure, Serbia, state capitalism, 一带一路, 金融化, 基础设施, 塞尔维亚, 国家资本主义, Nueva Ruta de la Seda, financiarización, infraestructura, Serbia, capitalismo estatal, финансиализация, инфраструктура, Сербия, государственный капитализм

## Abstract

The Belt and Road Initiative (BRI) has been theorised as a spatial fix to China’s overaccumulation problem, and as such, an implicitly productivist endeavour. This article opens up conceptual space to consider how historically and geographically mediated forms of financialisation have tempered the unfolding of the BRI in peripheral economies. Drawing on the Serbian post-socialist transition context, financialisation has been characterised by underinvestment and a persistent dependency on foreign, market-based capital inflows which have (1) precipitated state transformations to mobilise Chinese financing for BRI projects, strengthening the role of the state in industrial rejuvenation; and (2) created an institutional palimpsest conducive to non-productive forms of surplus value appropriation that demonstrates the hybridity of accumulation imperatives underlying the BRI.

## 摘要

超越空间修复:对‘一带一路’在塞尔维亚进行金融化的解读. *Area Development and Policy*. ‘一带一路’倡议被认为是解决中国资本过剩问题的空间修复方案,因此是一种隐性的生产主义的尝试。本文开辟了一个概念空间,以考虑历史上和地理上介导的金融化形式如何缓和了‘一带一路’在外围经济体的发展。从塞尔维亚后社会主义转型的背景来看,金融化的特点是投资不足和持续依赖基于市场的外国资本流入,这1)促使国家转型以调动‘一带一路’项目的中国融资,加强了国家在工业复兴中的作用,2)创造了一个有利于非生产形式的剩余价值占有的制度重写,表明了‘一带一路’背后的资本积累规则的混合性。

## RESUMEN

Más allá del remedio espacial: hacia una lectura financiera de la Nueva Ruta de la Seda en Serbia. *Area Development and Policy*. Se ha teorizado que la iniciativa de la Franja y la Ruta de China (Nueva Ruta de la Seda) es un remedio espacial de China a su problema de sobreacumulación y, por tanto, una tarea productivista implícita. En este artículo analizamos mediante un espacio conceptual de qué modo las formas de financiarización mediadas desde una perspectiva histórica y geográfica han atenuado el desarrollo de la Nueva Ruta de la Seda en economías periféricas. A partir de un contexto de transición en la era post-socialista de Serbia, la financiarización se ha caracterizado por una falta de inversión y una persistente dependencia de los ingresos de capital extranjero basada en el mercado, que ha (1) precipitado las transformaciones estatales para movilizar la financiación china de los proyectos para la Nueva Ruta de la Seda, y así reforzado el papel del Estado en el rejuvenecimiento industrial, y (2) creado un palimpsesto institucional que favorece las formas no productivas de la apropiación de la plusvalía y que demuestra la hibridez de los imperativos de acumulación subyacentes a la Nueva Ruta de la Seda.

## АННОТАЦИЯ

За пределами пространственного решения: к пониманию ‘Пояса и пути; в Сербии с учетом финансовых факторов. *Area Development and Policy*. Инициатива ‘Один пояс – один путь’ (BRI) теоретически рассматривалась как пространственное решение проблемы перенакопления в Китае и, в качестве таковой, как неявно продуктивистское начинание. Эта статья посвящена осмыслению того, как исторически и географически опосредованные формы финансиализации сдерживали развитие BRI в периферийных экономиках. Опираясь на сербский постсоциалистический переходный контекст, финансиализация характеризовалась недостаточными инвестициями и постоянной зависимостью от притока иностранного рыночного капитала, что (1) ускорило государственные преобразования для мобилизации китайского финансирования проектов BRI, усилив роль государства в промышленном возрождении, и (2) создало институциональный палимпсест благоприятствующий непроизводительным формам присвоения прибавочной стоимости, что демонстрирует гибридность императивов накопления, лежащих в основе BRI.

## 1. INTRODUCTION

In the decade since the establishment of the Belt and Road Initiative (BRI), a body of interdisciplinary literature has emerged that has interpreted its multifaceted dimensions as an umbrella phenomenon for China-led political and economic integration. However, there has arguably been greatest attention to the initiation of physical connectivity projects between China and BRI countries, namely land and maritime transport corridors, special economic zones, and other forms of built infrastructure necessary to support more intensive forms of regional economic integration, trade and investment (Bennett, 2016; Bucsky, 2020; Chubarov, 2019; Liu & Dunford, 2016; Liu et al., 2021; Vinokurov & Tsukarev, 2018).

Within the critical tradition, notably Marxist political economy and world systems theory, several scholars have sought to embed such projects in the historical and geographical materiality of global capitalist development, conceptualising the BRI as a spatial fix, a sprawling, fragmented infrastructure connectivity project intended to manage the exigencies of capital overaccumulation through spatial means (Apostolopoulou, 2020; Blanchard & Flint, 2017; Chacko & Jayasuriya, 2018; Mayer & Zhang, 2021; Summers, 2016; Zhang, 2017; Zhao, 2016).

These accounts have drawn attention to the productivist rationale of the BRI, as a state accumulation strategy to sustain the appropriation of surplus value through trade and commodity production, export China’s domestic capital and labour surplus and couple Chinese production and consumption to global supply chains in new markets (Lim, 2018; Schindler & Kanai, 2021). This article contributes to this body of literature by examining an overlooked aspect of the spatial fix: how financialisation as the historically and geographically mediated growth of private, market-based finance in economic and social life where surplus value is appropriated through financial, non-productive channels has contributed to the expansion of the BRI (Fine, 2013; Lapavitsas, 2013).

Drawing on the literatures on subordinate and peripheral financialisation (Alami et al., 2022; Becker et al., 2010; Bonizzi, 2013; Powell, 2013), this article develops a finance-sensitive reading of BRI expansion in the Serbian post-socialist transition context. The argument proceeds along two axes. First, financialisation has to varying degrees exacerbated low levels of productive investment and dependency on foreign, market-based capital inflows in peripheral states of the world economy, bringing about concomitant transformations of the state that have enhanced state capacity to mobilise Chinese financing for the establishment and implementation of BRI projects, and strengthen the role of the state more generally in industrial rejuvenation. Second, the legacy of financialisation in peripheral economies such as Serbia has created an institutional palimpsest shaping the investment rationale behind BRI projects. Contrary to the emphasis on industrial firms’ imperative of productive expansion, BRI investment projects have been mediated by an institutional context conducive to non-productive forms of surplus value appropriation, contributing to an increasing diversity of BRI financing models that reflects the hybridity of accumulation imperatives that underly it.

This article illustrates how these dynamics have played out in Serbia where post-socialist transition, location within the European periphery, as well as political economic subordination to the International Monetary Fund (IMF), the European Union (EU) and the United States (US) has brought about a unique trajectory of financialisation. First, this article shows that successive rounds of IMF- and EU-backed market liberalisation in Serbia have exacerbated problems of underinvestment and a persistent dependency on foreign market-based capital inflows that has given the incumbent government a rationale to turn towards China and the BRI. Serbia’s political economic China pivot has consequently incentivised the state to implement reforms designed to strengthen its capacity in mobilising Chinese financing for infrastructure development. Second, market liberalising policies that went hand in hand with the financialisation of the economy in the 2000s, including a generous foreign direct investment (FDI) regime and the introduction of private–public partnerships (PPPs), has mediated the investment rationale underlying BRI investment projects in Serbia such that they have been driven by an ad-mixture of both productive and financialised logics of accumulation.

Analysis is based on data collected from the field in Serbia between October and November 2020, where over 20 semi-structured interviews in Chinese and English with construction managers, government officials, consultants, translators, construction workers and other relevant personnel were conducted. Additional interviews in Beijing and Hong Kong in 2019 and 2020 provided further context. The analysis furthermore draws on an original database of ongoing infrastructure projects in Serbia, compiled in December 2021, supported with data from official sources including the Serbian Ministry of Construction, Transportation and Infrastructure, Chinese Ministry of Commerce, as well as media articles and secondary academic sources.

The remainder of the paper is structured as follows. The next section critically reviews the existing literature on the BRI and the role of financialisation therein. The article then follows with a discussion of how financialisation in peripheral economies pertains to the expansion of the BRI, before proceeding to a grounded discussion of how these dynamics have played out in Serbia where they have (1) catalysed BRI-friendly transformations of the state and (2) favourable FDI and PPP legislation has implied a hybridisation of accumulation logics underlying BRI projects. The article concludes with implications and ruminations for future research.

## 2. BRI: SPATIAL FIX AND BEYOND

The literature on the political economy of the BRI has mushroomed in volume and in the breadth and depth of analytical approaches since the elaboration of the five cooperation priorities first outlined in the official 2015 Vision and Actions on Jointly Building the Silk Road Economic Belt and the 21st Century Maritime Silk Road. Scholars have sought to theorise the BRI as a regional economic connectivity project in terms of its geopolitical, infrastructural, trade, financial, ecological, scientific and cultural dimensions (Bucsky, 2020; Gong, 2019; Lai et al., 2020; Liu & Dunford, 2016; Vinokurov & Tsukarev, 2018; Winter, 2021; Woods, 2022; Xiao & Parenti, 2022). Recent literature has increasingly opened up conceptual space to theorise the transnational, bottom-up, co-constituted and multi-scalar nature of its construction (Mayer & Zhang, 2021; Oakes, 2021; Oliveira et al., 2020; Paudel, 2021; Schindler et al., 2022b). These accounts have enriched attempts to draw out the broader capitalist dynamics of the BRI, and have added nuance to interpretations of its domestic political and geopolitical drivers, namely as an extension of the Go-West campaign to develop China’s comparatively underdeveloped inland provinces, demand for energy security as a consequence of growth, in improving relations within the Global South, and containing security risks in Eurasia (Zhao, 2016).

The literature that has theorised the BRI as a spatial fix stands out in the context of these approaches, where the expanding geographical boundaries of China-led regional connectivity is conceived of as a means to further capital accumulation through spatial expansion. In this vein, the BRI is a state accumulation strategy to manage the exigencies of overaccumulation, domestic economic slowdown, overcapacity in industry from overinvestment (decreasing returns to capital), rising wage levels, excess reliance on capital investment and depressed global demand on the heels of the global financial crisis (Apostolopoulou, 2020; Blanchard & Flint, 2017; Chacko & Jayasuriya, 2018; Summers, 2016; Zhang, 2017; Zhao, 2016). As a spatial fix, physical connectivity through built infrastructure such as road, rail, port, energy, communications, manufacturing facilities and special economic zones become the material basis to locate new markets for China’s surplus productive capacity and further couple Chinese industry to global supply chains (Lim, 2018; Schindler & Kanai, 2021).

While such a focus re-centres capitalism in understandings of the BRI, Mayer and Zhang (2021) caution that theorising the BRI as a spatial fix should not invite overly deterministic, structural readings. The state is always possessed of ‘heterogenous and shifting spatial strategies inside of global capitalism’, and thus ever in need of scholarly reinterrogation (Mayer & Zhang, 2021, p. 6). In official discourse, overproduction has always been highlighted as a key problem for the Chinese economy (Zhang, 2017). However, theorising the BRI as a spatial fix tends to reify its expansion as an exclusively productivist endeavour where the focus is on fixed capital formation, that is, built infrastructure necessary to develop the structures to support supply chains and worker mobility needed to sustain the territorial expansion of trade and production (O’Neill, 2013).

Yet, such a view is not the whole story. The BRI has unfolded within a larger set piece of capitalist development where historically and geographically mediated forms of financialisation have conditioned the expansion of the BRI as a purely productivist state accumulation strategy. The spread of financialisation where private, market-based finance-led growth has contributed to the appropriation of surplus value increasingly through financial markets, institutions, actors and practices over productive expansion have gained greater influence in peripheral, emerging and subordinate economies of the world market (Fine, 2013; Karwowski & Stockhammer, 2017; Lapavitsas, 2013), many of which overlap with the corridors of the BRI across Eurasia, South East Asia, Latin America, Africa and within China itself.

To be sure, there is a voluminous literature on the diversity of financing of BRI projects, but these accounts largely centre on the role of the Chinese state through the deployment of state capital via state-owned enterprises (SOEs) and state-owned financial institutions, to further productive expansion, including works that delve into the nature and implications of China’s bank-based debt-financing of infrastructure projects (Chin & Gallagher, 2019; Jepson, 2021), the creation of multilateral development financing institutions such as the Asia Infrastructure Investment Bank and the New Development Bank (Liu et al., 2020; Ly & Tan, 2020), and the emergence of a parallel China-led export-credit development financing regime to that offered by the existing liberal order (Chen, 2021). However, financialisation is analytically distinct from financing, which does not reflect how legacies of financial appropriation mediated by historical and geographical contexts have conditioned BRI expansion. The financing of BRI projects, albeit significant, is but one piece of the puzzle.

More tellingly, financialisation has unfolded within China itself, bringing about transformations of the state that have mediated the political economic underpinnings of the BRI. The 2000s marked China’s accelerated integration into the global economy. On the heels of the 2008 global financial crisis, the domestic economy became exposed to the effects of expanding liquidity, short-term speculation and massive concentrations of capital emanating from the advanced capitalist core where financialised forms of economic governance, such as the use of quantitative easing and other forms of monetary intervention, enabled vast amounts of liquidity to be channelled into the Chinese economy (Tsui et al., 2017). Such flows have been the material basis for the formation of transnational capitalist alliances, between the World Bank and pro-market reformers in China (Meulbroek, 2022; Weber, 2021), and SOE managers and private Chinese capitalists (Tsui et al., 2017), yielding an ‘interior bourgeoisie’ linked to both transnational and national circuits of capital that has accelerated the adoption of financialised forms of economic governance and defined in no small part the institutional foundations of how the BRI is financed and implemented (Chacko & Jayasuriya, 2018, p. 88; Gonzalez-Vicente, 2019; Oliveira, 2019). This includes corporate governance reforms that have institutionalised shareholder value in the management of state-owned assets and SOEs, the core industrial agents of the BRI. When BRI projects are defined as China-related projects either equity or loan financed in part by Chinese firms or financial institutions in countries whose governments have signed on to the BRI (Liu et al., 2020), SOEs account for over half of BRI projects contracted by number and over 70% by project value (Zhen, 2019). Chinese SOEs, including China’s biggest international construction contractors are nested in tiered ownership structures, such as China Road and Bridge Corporation, under the management of the primary state shareholder.

The state has also established sovereign wealth funds such as the China Investment Corporation, the brainchild of reform-minded officials and Wall Street Returnees, that is both investing China’s surplus savings into global financial markets driven by short-termist speculative financial practices alongside the world’s largest institutional investors but also, through its shareholdings in the Silk Road Fund, committed to reinvesting long-term capital and providing network resources to SOEs operating in BRI states (Liu & Dixon, 2022).

Lastly, the state has also issued guidelines encouraging Chinese contractors to pursue alternative forms of market-based financing to the bank-based models that have dominated BRI financing, where private partners can be enlisted to co-finance, manage or operate projects, that reflects a growing awareness of debt sustainability issues in BRI states (Brautigam, 2020; MOF, 2019; MOFCOM, 2020).

However, there has been insufficient attention to how financialisation has mediated the expansion of the BRI in peripheral economies outside China. To be sure, financialisation is far from a one-size-fits-all phenomenon, for some BRI states such as Belarus have maintained a strong state-led economic and social model that has seen the economy avoid the kind of market liberalising reforms that have taken place in other post-socialist transition contexts (Liu et al., 2021). At the same time, other BRI states as diverse as Serbia and Zambia have been subjects of financialisation. Both countries have been witness to high levels of foreign bank ownership, which tends to shift lending from small and medium-sized enterprises to the household sector, and that has done little to spur productive growth (Becker et al., 2010; Kvangraven et al., 2021).

These states constitute sites of conjuncture between two structuring political economic contexts wherein the BRI, largely conceived of as a productive state accumulation strategy, has been mediated by financialised modes of capital accumulation. Such sites warrant investigation because they are where the ‘political–economic contradictions of capitalism are particularly condensed’ (Anguelov, 2020, p. 8), and where an analytical lens that privileges the continuities, as opposed to the disjunctures, in capitalist development, can be particularly fruitful for understanding trajectories of global capitalism (Furlong, 2020). It is through an interrogation of financialisation as it has unfolded within these sites that we can understand the expanding contours of the BRI as part and parcel of global capitalism. This article opens up conceptual space to consider, explicitly, how, when and where financialisation has mattered for the material expansion of the BRI.

## 3. FINANCIALIZATION AND THE BRI IN PERIPHERAL ECONOMIES

The BRI is expanding within a context of world development where financialisation has already gained a foothold in peripheral economies. However, relative to their peers in the advanced capitalist core, the form and function of financialisation in peripheral economies takes on particular characteristics owing to their subordinate and dependent position in the global political economy that has been shaped by legacies of colonialism, imperialism, catch-up development and the secular tendencies of capitalist expansion emanating from the advanced capitalist core (Alami et al., 2022; Becker et al., 2010; Powell, 2013).^1^

A key driver of financialisation in peripheral economies has been the adoption of market-fundamentalist approaches to development promoted by the Washington Consensus, notably in Latin America, where market liberalising reforms were in large part (1) a policy response to the Volcker shock of the 1980s that crippled many states in the region that had borrowed from US banks and (2) subsequent intervention by the IMF as sovereign lender of last resort (Bonizzi, 2013; Carroll, 2017; Gabor, 2012; Mawdsley, 2018). The financialisation of development, predicated on US and IMF-led efforts in the 1980s to create a world market underpinned by neoliberal rules, has necessitated an ‘institutional fix’, that is, concomitant transformations of the state to mediate, consolidate and legitimise private, market-based finance-led growth in peripheral economies (Anguelov, 2020; Furlong, 2020; Meulbroek, 2022; Peck & Tickell, 1994).

One major consequence has been the decline in productive over financial investment by industrial firms due to the relative profitability of financial investment (Dunford, 2021), the rise of shareholder value orientations (Aglietta & Rebérioux, 2005; Lazonick & O’Sullivan, 2000), long-short speculative pressure from global institutional investors (Tsui et al., 2017), and high levels of foreign ownership in the domestic banking sector that have been observed in Central and Eastern Europe, Brazil, the Philippines and Zambia, which have given rise to the expansion of household credit at the expense of financing domestic industry (Bonizzi, 2013; Kvangraven et al., 2021). High levels of productive underinvestment reflect the inability of peripheral economies to realise long-term investment for industrial upgrading and real growth in gross domestic product (GDP) that reinforces their subordinate position in the international system.

Related to the issue of underinvestment, is a disproportionate shift towards foreign, market-based sources of capital to finance industrial development that has seen foreign investors appropriate a greater share of domestic surplus through short-termist speculative investment. These flows have been sustained by a set of policy instruments including capital account liberalisation, high interest rates, privatisation, and preferential tax and mobility regimes (Becker et al., 2010; Bonizzi, 2013). Bank-based forms of development finance and official development assistance have lost ground to private market-based capital in the political arena where it is believed that varying forms of PPPs can be leveraged for infrastructure development (Mawdsley, 2018). In PPPs, the infrastructure may be packaged into various products (assetised) that maximise short-term revenues and sold to the highest bidder on commodity markets (O’Neill, 2013). Private partners may leverage their knowledge and expertise in financialising infrastructure to maximise short-term profitability over long-term developmental gains to the advantage of transnational investors.

These dual dynamics have had two implications for the material expansion of the BRI. First, the BRI offers patient capital for infrastructure development and industrial investment in peripheral economies that is an alternative to Washington Consensus-backed forms of market-based development where states are either faced with development aid tied to complex bureaucratic procedures and governance conditionalities, commercial lending offered at market rates, or PPPs in the case of infrastructure, beholden to short-termist investor logics (Chen, 2021; Kaplan, 2021; Mawdsley, 2018). Empirically, this is supported by the high incidence of overlap between peripheral economies that have been subject to IMF conditional lending and BRI states. Drawing on data provided by Kentikelenis et al. (2016), all the states identified as subject to the highest overall IMF conditionality burdens in the period between 1985 and 2014, including Romania, Pakistan, Tanzania, Kyrgyzstan and several West African countries, have signed on to the BRI.

In contrast to the Washington Consensus, China’s BRI financing, whether equity or debt, has been conceptualised as state-coordinated due to the stronger role of the state, as opposed to international private capitalists via the market, in the financing and implementation of BRI projects. This is particularly pronounced in infrastructure development where projects typically follow from memoranda of understanding (MOUs) and other framework agreements between China and BRI states, often concluded at the highest levels of state. In addition to Chinese SOEs who, as previously elaborated, have been the core industrial agents on BRI projects, Chinese state agencies such as the Ministry of Commerce play a coordination role in BRI projects, matchmaking regions with projects, firms and investors, and who work intensively with parallel agencies in BRI states to realise projects (Liu & Dixon, 2022).

The more visible role of the state is reflected in the ratio of China’s sovereign lending to BRI states, which accounts for 66% of China’s total outward FDI stock to these countries (Liu et al., 2020). Moreover, of all outstanding loans in BRI countries, 70% is held by China’s two main development banks, China Development Bank and China Export Import Bank, which lend at semi-commercial rates, and where projects are usually evaluated over an industrial cluster or geographical region (Chin & Gallagher, 2019; Liu et al., 2020). As a consequence, many peripheral economies have been able to mobilise Chinese development finance for BRI projects where they cannot secure financing elsewhere, especially when they constitute key logistical corridors between China and new markets.

However, BRI projects have required an institutional fix inverse to that of the Washington Consensus wherein the strong role of the Chinese state in the financing and implementation of projects has necessitated a similar alignment in BRI states. Lending in many cases has been tied to the selection of Chinese SOEs as lead contractors. In the case of engineering, procurement and construction (EPC) or turnkey projects, where the BRI state need only ‘turn the key’ once construction is completed, the approval of the loan agreement is conditional on the approval of the commercial agreement with the contractor, transforming them into EPC+F (finance) projects (Liu & Dixon, 2022).

Existing rules on public procurement such as the Agreement on Government Procurement promulgated at the global level and backed by Washington Consensus governance conditionalities require open tender for the selection of construction contractors (World Trade Organization [WTO], 2022). The financing conditionalities on BRI projects would require an amendment of existing law in order for EPC+F projects to legally bypass the competitive tender process. Paralleling the rise of the infrastructure state in Laos, Nepal and Ethiopia where they have introduced political and institutional reforms that bolster state capacity to mobilise Chinese development finance for BRI projects (Chen, 2020; Paudel, 2021; Schindler et al., 2022), financialisation has essentially provided material and ideological legitimacy to peripheral states to pursue alternative models of development beyond the Washington Consensus, bringing about transformations of the state, such that it has come to play a greater role in economic development.

Second, at the same time that there has been a strengthening of the role of the state in the economy, there has also been continuity where financialisation-linked market liberalising reforms in peripheral economies have been sustained by alliances between international private and domestic capitalists despite the state transformations that have been initiated to accommodate the more visible role of the state arising from China-backed projects.

Policies that have attracted foreign private investors are also a boon to Chinese SOEs on BRI projects. Financialisation in peripheral economies essentially creates an institutional palimpsest that inserts the productive rationale of the BRI into an institutional landscape conducive to financialised logics of surplus value appropriation, encouraging both productive investment and profit-seeking through financial, non-productive channels on BRI projects. Precedent illustrates how Chinese SOEs in the context of their global integration have been incentivised to decouple from the productive rationale of the BRI. First, SOEs have channelled capital into the financial services sector, including leading investment banks, accountancy firms and law firms that have enculturated SOEs to financial best practices that enhance profitability not through productive expansion but by economising on invested capital that constitutes a form of shareholder value maximisation (Aglietta & Rebérioux, 2005). This includes outsourcing through temporary contracts, seeking out tax-friendly FDI destinations, and adopting practices such as roundtripping through offshore subsidiaries where they can enjoy the tax and regulatory benefits afforded to foreign investors in China (Liu & Dixon, 2021; Wójcik & Camilleri, 2015). Temporary subcontracting is standard practice on infrastructure projects, while prominent SOEs engaged on BRI projects such as China Harbour Engineering Company have adopted accounting practices such as the use of offshore tax havens in Delaware and the Bahamas to avoid double taxation (Gonzalez-Vicente, 2019; Liu, 2021).

Second, SOEs already demonstrate their capacity to maximise the interests of the firm over the developmental rationale of the BRI. SOEs have made alliances with domestic capitalists in BRI states to realise projects that reflect more the competition and threat of moral hazard within the state than a productive use of state capital (Jones & Zou, 2017; Shi, 2015). Guangxi provincial state and capital interests formed a partnership with Malaysia’s Pahang state in 2013 to develop port, rail and road infrastructure only to be followed by a similar project along the same peninsula developed between Guangdong province and Malacca state (Jones & Zeng, 2019). The conjuncture of financialisation- and BRI-linked state transformations in peripheral economies constitutes a structuring political economic context that reflects the hybridity of capital accumulation logics underpinning the expansion of the BRI.

## 4. FINANCIALIZATION IN POST-SOCIALIST SERBIA

One site of conjuncture where the hybridity of productive and financialised accumulation logics has played out is Serbia where historical trajectories of development rooted in the transition from socialist Yugoslavia and post-war reconstruction have given rise to financialisation, characterised in particular by underinvestment in production and dependency on foreign, market-based capital inflows, in the post-socialist context.

The introduction of market-liberalising reforms was precipitated by a period of economic decline after a post-war mid-century growth boom. Foreign capital had already become a major pillar of growth as early as 1965 (Radenković, 2017). Paralleling macroeconomic conditions in Latin America, high levels of foreign borrowing plunged the country into debt crisis on the heels of the Volcker Shock and the concomitant rise in global interest rates in 1979, precipitating the conclusion of seven standby arrangements with the IMF that led to further liberalising reforms promoting marketisation, internationalisation, state rollback, governance reforms and the wholesale abandonment of socialism beginning in the late 1980s (Mikuš, 2016). Thereafter, the period from 1990 to 2000 was characterised by the decomposition of socialist Yugoslavia, civil war, hyperinflation and economic isolationism, though prior to the severing of ties in March 1999, the US had already sought to influence a pro-liberalising reform agenda through programmes funded by the National Endowment for Democracy, a US-funded non-governmental organisation subject to congressional oversight (Scott & Steele, 2005).

The Milošević era came to an end in 2000 after intervention in Yugoslavia by the North Atlantic Treaty Organisation. Thereafter successive waves of IMF and EU accession-linked financial liberalisation led to underinvestment in productive capacity, overexposure and dependency on foreign capital inflows, as well as high levels of foreign debt and ownership in the financial sector, features that have come to characterise financialisation in the post-socialist Eastern European periphery (Ban & Bohle, 2021; Becker et al., 2010; Nelson, 2020).

The EU in particular exercises structural power in the Western Balkan states of Albania, Bosnia and Herzegovina, Kosovo, Montenegro, North Macedonia, and Serbia via the Stabilisation and Association Agreement, which Serbia signed in 2008. The agreement grants accession states access to EU assistance and support programmes but entails the adoption of governance reforms that will eventually align with the regulatory frameworks of the EU (Lađevac, 2020; Pavlićević, 2019). Combined with post-liberalisation regional market integration, political and economic proximity to the EU has put pressure on accession states to adopt a strictly market correcting role in economic governance, accelerating dynamics of financialisation in the region.

Financialisation has, in the context of rapid market liberalisation in the 2000s, been linked to low levels of productive investment. Serbia first witnessed a growth boom, fuelled by global savings and a high interest rate, that led to a dynamic of import-led development. Combined with an appreciating exchange rate due to the rapid inflow of capital, this prompted a growing structural imbalance in the economy (Radenković, 2017). Investment largely flowed into the FIRE sectors (finance, insurance and real estate – FIRE) as a result of the restructuring and privatisation of the banking sector. In the period 2000–12, Serbia evidenced the lowest share of total investments to GDP in the Western Balkans and of the top 10 foreign investments in Serbia, only two have been greenfield projects (Radenković, 2017, p. 33). Equity inflows were largely used to buy up former state-owned financial institutions, helping to shore up the power of international private and domestic capitalists (Radonjić, 2018). Highly unusual for developing countries, by 2008 financial services amounted to 46.9% of FDI, above the global participation rate (Becker et al., 2010; Radenković, 2017).

Financialisation has persisted in the post-financial crisis era. If anything, Serbia’s current account deficit and worsening international investment position exacerbated the effects of the crisis. The dinar steadily depreciated, sparking concerns that foreign banks would transfer funds out of Serbia, precipitating subsequent rounds of IMF assistance and structural adjustment (Becker et al., 2010). Due to the dominance of the FIRE sectors underpinned, in particular, by transnational alliances between domestic and EU capitalists, Serbia’s post-crisis experience emulates similar failures to de-financialise other economies in the EU periphery (Ban & Bohle, 2021). Expansionary monetary policy in Serbia and as a result of quantitative easing measures enacted by the Federal Reserve and the European Central Bank saw an increase in bank lending, but that flowed primarily to the household sector as banks, largely foreign owned, sought to avoid credit risk from real economy lending. Between 2005–2015, growth in real economy money supply accounted for only 93 billion dinars, versus 179 billion in the household sector, fuelling a chronic state of productive underinvestment (Radenković, 2017).

Financialisation in Serbia has also been characterised by a dependency on foreign, market-based capital inflows that has been an outcome of market liberalisation. In terms of monetary and exchange rate policy, the persistence of capital inflows has been sustained by a high benchmark interest rate, which has encouraged speculation since foreign investors sell foreign currency to purchase state securities in dinars. Although this leads to a loss of liquidity, the state has sought to maintain this dynamic since the purchase of dinars is used to maintain the exchange rate (Radenković, 2017).

There have also been successive waves of fiscal policy reforms to mobilise foreign capital that, combined with the privatisation of the banking sector, channelled more FDI into FIRE sectors over real economy assets. Beginning with the 2002 Law on Foreign Investment that equalised the rights of foreign and domestic investors, the state has since established a host of subsidy and tax relief measures for foreign investors. According to the Decree on Rules for State Granted Aid issued in 2014, large-scale investors could potentially receive up to 50% of the total value of their investment, and foreign investments that meet certain conditions are exempt from corporate tax for the first 10 years of operation (Radenković, 2017).

Lastly, the state has sought to use financialised forms of infrastructure development to spur productive growth. The Law on Public Private Partnerships and Concessions was introduced in 2011 and a nine-member PPP Commission established in 2012, providing an institutional framework for private, market-based infrastructure procurement. Between 2012 and 2020, the PPP Commission approved 169 projects, totalling €3 billion in value (Public Procurement Administration, n.d.). These figures are modest, relative to the value of China-financed EPC+F projects, but over 25 subnational city and municipal authorities have entered into at least one PPP or concession contract (Gazivoda, 2021), signalling a maturing of the PPP model in Serbia as a model of infrastructure financing.

## 5. THE BRI AND STATE TRANSFORMATION IN SERBIA

As the preceding section has outlined, financialisation has taken on unique features in the context of Serbia’s post-socialist transition and has laid the foundations for Serbia to sign on to the BRI. While there has been a strong geopolitical rationale for Serbian support for China-led connectivity in the region, especially as a means to balance EU, US and Russian influence, it was not until after the global financial crisis in the face of Serbia’s rapidly declining economic fundamentals that political economic relations with China rapidly picked up. Materialised in the debate around monetary stability (curbing inflation) versus developing the export base (exchange rate depreciation), the question of how to rebalance the economy was a key election issue in the aftermath of the financial crisis (Becker et al., 2010).

Although the BRI was not officially launched until 2013, Serbia had already made an official pivot to China by signing the Agreement on Comprehensive Strategic Partnership with China in 2009 on the heels of the financial crisis, marking Serbia as China’s only strategic partner in Southeast Europe at the time (Lađevac, 2020). Subsequent to the signing of the strategic partnership, Serbia joined the 16+1 cooperation mechanism initiated by China in 2012, and that has become integrated with China’s diplomatic efforts to promote the BRI in 16, later 17, Central and Eastern European states (Jojić, 2017). Serbia is a key BRI state in the context of the proposed China–Europe Land–Sea Express Route, a logistical corridor enabling the transport of goods by sea and rail from China via the Greek Port of Piraeus through the Western Balkans and Hungary into Western Europe (Zweers et al., 2020). Moreover, the financial crisis gave Serbia an additional reason to turn towards China for post-war industrial reconstruction and development since EU funds promised as part of the Stabilisation and Association Agreement had failed to materialise in the context of the EU’s ongoing liquidity issues (Lađevac, 2020).

The strategic partnership precipitated a series of state transformations in Serbia that have strengthened the capacity of the state to establish BRI projects. First, China and Serbia signed a landmark treaty, the 2009 Agreement on Economic and Technical Cooperation in the Area of Infrastructure. This was the first major institutional reform to bolster the role of the state, as opposed to the market, in mobilising Chinese financing for industrial development. The agreement ties Chinese financing to construction, clearing the need for pre-selection and open tender by the lead contractor on public infrastructure projects, allowing Chinese contractors to bypass the hurdle of competitive pre-selection where they would have to compete against other, mostly European, contractors (Rogelja, 2020). Moreover, the treaty stipulates that contractor and subcontractor selection procedures be outlined in the (confidential) commercial contract, thus protecting the allocation of loan funds from public scrutiny. The treaty also empowers any state entity, including banks and SOEs, to act as representatives of China in negotiations with the investor, the Serbian government or Serbian SOEs. In the realm of labour, the treaty grants immigration clearance exceptions for construction staff, and more generally casts a legal grey area exempting Chinese labour workers from Serbian labour law (Liu, 2020).

Second, both the 2009 agreements laid the foundations for the first major Chinese infrastructure project in Europe to be undertaken in Serbia. Valued at €170 million, the Pupin Bridge in Belgrade was an EPC+F project launched in 2011 that set the precedent for China financed EPC+F projects in Serbia going forward. As of year-end 2021, there were 11 active infrastructure projects tied to Chinese contractors that had progressed beyond MOU stage (Table 1). Six were confirmed EPC+F projects, totalling over €2 billion in lending. This number will potentially increase to seven, totalling more than €6 billion in outstanding Chinese loans if the Belgrade metro is loan financed by China.

**Table 1.** Active Belt and Road Initiative (BRI) infrastructure projects in Serbia, December 2021. Project size, financing terms and status are updated as of March 2023.

Name	Financing model	Project size	Financing terms	Investor/operator	Main contractor(s)	Status
Belgrade–Nis railway	EPC	€2.2 billion	€500 million loan from the European Bank for Reconstruction and Development. €1.1 billion loan from the European Investment Bank and €550 million in EU grants	Serbia Railways Infrastructure	China Road and Bridge Corporation (last rumoured)	Framework agreement signed in 2018; however, 100% EU financing places Chinese involvement in doubt
Belgrade–Subotica railway (section of the Belgrade–Budapest railway)	EPC+F	€1.243 billion	€1 billion loan from the China Export Import Bank	Serbia Railways Infrastructure	China Railways International and China Communications Construction Company	Works began June 2018
Belgrade Bypass highway	EPC+F	€657 million	Loan worth €184 from the China Import Export to cover 85%	Roads of Serbia and the Ministry of Construction, Transport and Infrastructure	Power China and Azerbaijani firm Azvirt	Works began September 2018
Kostolac B thermal coal-fired power plant	EPC+F	€822 million	Loan worth €443 million from the China Export Import Bank loan to cover 85%	Elektroprivreda Srbije	China Machinery and Engineering Corporation	Expected completion in 2023
Kolubara thermal power plant	EPC	€385 million	Unknown	Elektroprivreda Srbije	Power China (last rumoured)	Preliminary deal signed in early 2020, but there are unofficial reports that the plant is being decommissioned
Fruska Gora tunnel	EPC+F	€660 million	China Export Import Bank loan. Amount undisclosed	Putevi Srbije (Roads of Serbia)	China Road and Bridge Corporation	Works began May 2021
Industrial Park in Borca	FDI: Greenfield joint venture	€300 million	Investment worth €220 million from the China Road and Bridge Corporation	Joint venture between Government of Serbia and China Road and Bridge	China Road and Bridge Corporation	Agreement signed May 2019
TE-TO Pancevo thermal power plant	EPC	€180 million	Majority Gazprom financed	Joint venture between Centroenergoholding (sub of Gazprom Energoholding and Serbian oil firm Nis)	Shanghai Electric Group	Trial operation and commercial exploitation in 2022
Preljina–Pozega highway	EPC+F	€450 million	Exim loan of €381 million	Putevi Srbije (Roads of Serbia)	China Communications Construction Company	Works began May 2019
Belgrade Metro	EPC(+F)	€4.4 billion	Donation from the French government of €8.3 million. China is expected to cover the financing for the Chinese portion of construction	City of Belgrade, Ministry of Construction Transport and Infrastructure, PUC Belgrade Subway and Train	Power China and Alstom	Works began Nov 2021
Resnik–Velika Plana rail	EPC	€340 million	To be determined	Serbia Railways Infrastructure	China Road and Bridge Corporation	Framework agreement concluded in 2019

Note: Only those projects that have progressed beyond the memorandum of understanding (MoU) stage are included, that is, at the very least where preliminary agreements identifying the key parties, investor and main contractor, have been signed. EPC, engineering, procurement and construction; +F, plus finance.

Sources: Author. Documentary sources are available on request.

In the 10 years since establishment of the BRI, the political impetus to support subsequent transformations of the Serbian state has been sustained. As President Tomislav Nikolić expressed in an official visit to Beijing in 2017, more BRI projects would go ahead so that Serbia could ‘fit much better within the Belt and Road Initiative’ (Mu, 2017). This has been reflected in the introduction of further legislative reforms empowering the state to initiate infrastructure projects. In February 2020, the Serbian government introduced 13 new laws and amendments concerning the implementation of public infrastructure works, in particular a law on highway and railway infrastructure that authorises the state to expedite the typically complex land expropriations necessary for the construction of public works, including all pipeline projects not yet initiated (Gazdic & Jandrić, 2020). Such legal reforms strengthen the capacity of the state to deliver projects, though this has come at the cost of democratic due process, notably in the relocation of Roma settlements (interview with an SOE manager, 21 October 2020).

These state transformations sit at odds with the market-based regulatory frameworks that EU accession states are required to implement as a condition of EU membership. In fact, the amendment of existing legislation to bypass competitive tender on public procurement projects violates EU regulation and has been criticised by the EU, as in the case of the Belgrade–Budapest railway where Hungary, as one of the main investors, is an EU member state (Rogers, 2019). The transformations of the state that have mobilised Chinese finance for BRI projects in Serbia therefore signal a deliberate divergence from path dependent trajectories of financialisation that have unfolded as a result of market liberalisation.

## 6. BRI IN CONTEXT: FDI AND PPPs IN SERBIA

At the same time that there has been divergence, there has also been continuity where the financialised policy terrain that has sustained market-based sources of foreign capital flows has brought about a hybridisation of accumulation logics underlying BRI projects in Serbia. The FDI regime and the PPP framework are of particular interest in this respect.

To begin with, the major BRI-linked investments have been brownfield projects,^2^ as opposed to greenfield projects that can generate new productive capacity, employment and industrial spillover effects, where investors can still take advantage of Serbia’s generous FDI regime that has largely facilitated the flow of foreign capital into privatised former state assets in the FIRE sectors without the greater financial risk and capital outlay required of greenfield investments. In terms of infrastructure, there is only one ongoing greenfield project, the industrial park in Borca, a joint venture co-financed by the Government of Serbia and China Road and Bridge, though, even there, there has been little progress on the project since the initial agreements were signed (Table 1).

The highest profile BRI-linked investment project has been the Smederevo steel plant. Formerly owned by US Steel, the Smederevo steel plant was acquired by Hesteel Group, a Hebei provincial-level SOE, in 2016, after the Serbian government reacquired the highly indebted plant from US Steel for €54 million with another subsequent capital injection of €120 million (HBIS Group, n.d.). As the first FDI project in the country mentioned in official discourse on the BRI, the plant has been touted as a symbol of the ‘iron friendship’ between China and Serbia (State Council, 2018). Less covered in official media is how the project has been able to take advantage of the existing FDI regime. The Serbian state has granted Hesteel highly favourable tax and labour subsidies, including tax exemptions for up to the first 13 months of employment of Chinese expatriated workers (interview with an SOE consultant, 3 November 2020). The investment has been built on FDI-favourable legislation intrinsically linked to processes of market liberalisation and financialisation in Serbia, yet it still constitutes a form of productive reinvestment that has kept in operation one of the country’s biggest exporters (State Council, 2018).

The other major FDI investment in official BRI discourse has been the acquisition of the RTB Bor copper mining complex by publicly listed Zijin Mining, in which the municipal city of Shanghai holds a quarter stake (Ministry of Foreign Affairs, 2022). Zijin acquired 63% of the highly indebted RTB Bor copper mining complex in 2018 from the government of Serbia (Zijin Mining, 2018). This is a project for which Serbia’s FDI regime proved decisive. The terms of the deal were that the government was to cancel a large sum of debt (rumoured to be up to €5.8 billion) owed to central and municipal authorities, in addition to tax exemptions on the first 10 years’ revenue stream and imported construction materials used to upgrade existing facilities, terms that clinched the acquisition for Zijin (N1, 2019; interview with an SOE manager, 23 October 2020). In addition to state-owned investments, private Chinese investors, less covered in official discourse, have also taken advantage of the regime. One study estimates in the case of Mei Ta, a private Chinese company that signed an agreement with the Serbian government in 2016, the state’s outlay of €21 million in employment subsidies and tax breaks far exceeded the €10.1 million paid in taxes and contributions over the same five-year period (Radenković, 2017).

In the second instance, the PPP framework in Serbia has encouraged the development of a greater diversity of BRI financing forms beyond EPC+F and EPC. The Kovin Energy Complex, a proposed underwater coalmine and thermal power plant in Kovin, represents one of the first forays into market-based forms of BRI financing in Serbia. The ongoing financier of the project was NIS whose shareholders comprise a conglomerate of Russia’s state-owned Gazprom, the Serbian government and various minority shareholders (NIS, n.d.). At the time, Gazprom was facing funding cuts in the wake of the Crimea sanctions and had wanted to take a backseat in the development of the Kovin project. Huadian, one of China’s five largest power generation firms that has been involved in BRI projects in Indonesia, Romania and Kenya, came on board as the majority owner (Energy System Integrator, 2016; interview with a private consultant, 10 March 2021) in what was proposed as a build–operate–transfer (BOT) project, a common form of PPP that entails equity ownership for the contractor (Eder & Mardell, 2018; Skidmore, 2022; Tritto, 2021).

Kovin would have been a departure from the more state-coordinated financing models that predominated in BRI projects at the time. Construction of the Pupin Bridge in Belgrade only broke ground in 2011. When the Kovin project was brought to Huadian in 2012, EPC+F projects were the norm.

The plan was to emulate the business model of private firms in the energy market such as EFT Group, an established Serbian private firm that had developed several coal-fired power plants in Bosnia that had adopted the BOT model (EFT, n.d.). Huadian and Gazprom had hired a Serbian engineering management consultancy who advised the investors to take advantage of the new PPP law. This intersected with state efforts to attract international energy companies to co-develop energy infrastructure sorely in need of upgrading since the energy market in Serbia is characterised by mostly public ownership, outdated infrastructure and a high dependency on coal, which accounts for 70% of Serbia’s energy mix (Petrikić, 2015). However, the consultancy proposed that the state should not be the end buyer of the electrical energy, and rather emulate the success of EFT Group in selling to European electrical energy trading companies where higher prices could be fetched than in the regulated domestic market (interview with a private consultant, 10 March 2021). In contrast, other BRI state-coordinated energy projects in development at the time such as the Kostolac coal-fired power plant were part-financed by the Serbian state via the national power utility Elektroprivreda Srbije (Table 1).

Although the Kovin project has stalled, it represents one of the first attempts to diversify BRI finance in an institutional context predisposed to financialised forms of infrastructure development. Huadian had taken seriously the possibility of selling on the private market, having gone as far as signing an MOU with the Swiss energy trader, Alpiq (interview with a private consultant, 10 March 2021). Buoyed by recent legislation promoting PPPs, the Kovin project was initiated by a transnational alliance between domestic and international state and private capitalists that had induced Huadian to seek out alternative sources of market-based finance. It represents a hybridisation of accumulation logics underpinning the BRI wherein infrastructure projects can serve both productive expansion and act as a channel for private surplus value appropriation

## 7. CONCLUSIONS

This article enriches current debates on the broader capitalist dynamics of the BRI by developing a finance-sensitive reading of how it has unfolded in peripheral economies. In doing so, it builds on existing literature that has sought to conceptualise the BRI as a transnational spatial fix (Apostolopoulou, 2020; Blanchard & Flint, 2017; Chacko & Jayasuriya, 2018; Summers, 2016; Zhang, 2017; Zhao, 2016). Where such accounts theorise the BRI as implicitly production-based, this article has opened up conceptual space to consider how the financing and implementation of BRI projects in peripheral economies such as Serbia has been mediated by historically and geographically informed processes of financialisation.

First, financialisation in Serbia has taken place within the context of post-socialist transition and post-war reconstruction where Washington Consensus-style market liberalisation has brought about productive underinvestment and high dependence on market-based capital inflows, symptoms characteristic of peripheral economies that reinforce their subordinate position in the global political economy. Financialisation has consequently been the political and economic backdrop to a series of state transformations that have bolstered the capacity of the Serbian state to mobilise Chinese finance for BRI projects for industrial rejuvenation, including a foreign policy pivot towards China, discretionary legislative reforms that empower the state to expedite China-financed infrastructure projects, and the initiation of a large number of state-coordinated BRI projects.

Second, path-dependent market liberalising policies that have characterised the financialisation of the Serbian economy have created an institutional palimpsest conducive to both financialised and productive investment on BRI projects. At the same time that a generous FDI regime and the promotion of PPPs have attracted short-termist speculative foreign investment into the Serbian economy, these policies have also been a boon to BRI projects that have been able to use the existing policy landscape to achieve discretionary tax and payroll gains. The highest profile FDI investments, the Smederevo steel plant and the Bor copper-mining complex, have both been brownfield projects linked to the large-scale privatisation of former state-owned assets in the FIRE sectors as opposed to greenfield projects that generate new industrial capacity. Lastly, the Kovin project illustrates how institutional conditions favourable to the establishment of PPPs have encouraged a greater diversity of BRI financing forms that have the potential to empower the private market in assetising and trading infrastructure.

Although the BRI is touted as a productivist state accumulation strategy, fragmented configurations of state and market power have clearly informed its unfolding. The BRI has come to symbolise the more visible role of the state in carving out policy space for industrial development, but as this article has shown, its expansion has been underpinned by a hybridisation of productive and financialised accumulation logics. Together with the resulting state transformations that have empowered the Serbian state, the insights generated here reinforce the value of an analytical lens that privileges continuity, as well as disjuncture, in understanding capitalist development as an uneven and *combined* process (Alami & Dixon, 2021; Dunford et al., 2021; Furlong, 2020; Rolf, 2021).

Sustained engagement with the literature on subordinate and peripheral financialisation would be a productive next step in thinking through the BRI as capitalist continuity (Alami et al., 2022; Lapavitsas & Soydan, 2022). The BRI is often discussed in relation to the big picture question of whether Chinese development finance presents an alternative paradigm to the Washington Consensus (and the emerging Wall Street Consensus) capable of renegotiating peripheral economies subordinate position in the global political economy. However, the extent to which the BRI represents a departure from the status quo requires an articulation of the highly unequal relations of power that reproduce the global financial system. Subordinate financialisation offers a relational framework to explain why so many states have signed onto the BRI and how China is shaping contemporary trajectories of development.

The intersection of financialisation and the BRI as it has played out in Serbia resonates with the turn towards the private market to aid development. China is showing signs of development fatigue since the state has had to contend with multiple solvency crises among distressed BRI debtors (Kynge et al., 2022). While market-based forms of finance such as PPPs are still relatively nascent in the context of the BRI, they are an attractive option for China and peripheral economies who may turn increasingly to private finance for liquidity (Gabor, 2021; Mawdsley, 2018; Schindler et al., 2022aa), reinforcing calls for critical scrutiny of the extent to which the financialisation of development can truly shore up the industrial base and rebalance indebted peripheral economies.

The approach taken in this article amplifies the need to constantly reassess the BRI vis a vis the concrete processes and social relations constituting the historical and geographical development of capitalism (Alami & Dixon, 2021), and how it is through the interrogation of intersections or sites of conjuncture of structuring forces (Anguelov, 2020) that the concrete unfolding of capitalist development gains clarity.

## NOTES

1. Several currents in the literature articulate financialisation in peripheral economies. Although they draw from different theoretical traditions, these literatures have been deployed interchangeably in theoretically generative ways because they share a common agenda in drawing out financialisation in peripheral economies as a distinct and, more importantly, derivative counterpart to financialisation in advanced capitalist economies, foregrounding the unequal power relations that inform financialisation in geographies beyond the core (Lapavitsas & Soydan, 2022).

2. A BRI investment project is defined here as projects entailing FDI equity financing from Chinese investors, as opposed debt financing of infrastructure projects.
